# Structural and dynamical investigation of histone H2B in well-hydrated nucleosome core particles by solid-state NMR

**DOI:** 10.1038/s42003-023-05050-3

**Published:** 2023-06-24

**Authors:** Xiangyan Shi, Bhuvaneswari Kannaian, Chinmayi Prasanna, Aghil Soman, Lars Nordenskiöld

**Affiliations:** 1 Department of Biology, Shenzhen MSU-BIT University, Shenzhen, Guangdong Province China; 2 School of Biological Sciences, Nanyang Technological University, Singapore, Singapore; 3 NTU Institute of Structural Biology, Nanyang Technological University, Singapore, Singapore; 4 Present Address: Department of Physiology and Biophysics, Case Western Reserve University, Cleveland, OH USA

**Keywords:** Molecular conformation, Gene regulation, Solid-state NMR

## Abstract

H2A-H2B dimer is a key component of nucleosomes and an important player in chromatin biology. Here, we characterized the structure and dynamics of H2B in precipitated nucleosome core particles (NCPs) with a physiologically relevant concentration using solid-state NMR. Our recent investigation of H3-H4 tetramer determined its unique dynamic properties and the present work provides a deeper understanding of the previously observed dynamic networks in NCP that is potentially functionally significant. Nearly complete ^13^C, ^15^N assignments were obtained for H2B R30-A121, which permit extracting unprecedented detailed structural and amino-acid site-specific dynamics. The derived structure of H2B in the well-hydrated NCP sample agrees well with that of X-ray crystals. Dynamics at different timescales were determined semi-quantitatively for H2B in a site-specific manner. Particularly, higher millisecond-microsecond dynamics are observed for H2B core regions including partial α1, L1, partial α2, and partial L3. The analysis of these regions in the context of the tertiary structure reveals the clustering of dynamical residues. Overall, this work fills a gap to a complete resonance assignment of all four histones in nucleosomes and delineates that the dynamic networks in NCP extend to H2B, which suggests a potential mechanism to couple histone core with distant DNA to modulate the DNA activities.

## Abstract (web summary)

This solid state NMR study of histone H2B provides insight into its structure and dynamics within the context of the nucleosome core particle.

## Introduction

The disc-shaped nucleosome core particle (NCP) serves as the basic unit of chromatin and is organized by the DNA wrapped around a histone octamer that is a complex of histone H2A, H2B, H3, and H4 proteins^1–4^, which is connected by linker DNA to form chromatin. The histone octamer provides a stable platform and, meanwhile, preserves plasticity to support DNA regulation. The modulation of DNA activities is achieved by various mechanisms including post-translational modifications (PTMs)^5–8^, incorporation of histone variants^9,10^ and interactions with effector proteins^11,12^. With the recent advances in structural biology techniques, substantial atomic-resolution structures of nucleosomes and nucleosome-protein complexes have been recently solved, which expands our understanding of gene regulation^1,13–16^. In addition, the dynamics of nucleosomes and sub-nucleosomes has been recognized as essential to chromatin biology^17–20^. Chromatin is a highly dynamic system at both the microscale and mesoscale levels, which is well modulated by the regulation factors in order to maintain cellular homeostasis^17,19^. Recent studies by NMR, MD, and FRET have reported microscale dynamics with high spatial and temporal resolution for nucleosomes^18,21–27^, revealing functional dynamical features of histones and DNA in the complexes. In addition, phase separation phenomena have been investigated for chromatin for many decades^28–30^, and recently chromatin liquid-liquid phase separation behaviors attracted much attention as it potentially describes new mechanisms of DNA regulation although controversial in-vivo and in-vitro results have been obtained for this biophysical property^31–33^, awaiting for further investigation. Solid-state NMR (SSNMR) has recently emerged as one of the high-resolution techniques that is capable to elucidate the dynamics and conformational assembly in chromatin biology^22,34–37^. For example, the SSNMR study of nucleosome arrays harboring the histone H4 aa K20 mono-methylation (H4K20me1) reveals that this PTM enhances the dynamics of histones and reduces the compaction of nucleosome arrays, which explains its abundance in transcription active regions^38^. Our previous investigation of nucleosome dynamics elucidated the existence of dynamic networks in the H3-H4 tetramer formed by a number of residues^39^. Those networks enable the coupling between the histone core regions with DNA and may transmit epigenetic signals from the core regions to the afar DNA sites. Similar functional dynamics of nucleosomes was recently reported by an MD study conducted a 15-microsecond simulation of a NCP^23^. The existence of dynamic networks is also suggested by a solution-state NMR study of NCPs harboring acetylation sites in the H3 and H4 N-tails^40^. However, the H2A-H2B dimer is another key component in chromatin biology, and the full picture of its dynamic properties in the nucleosome core is still lacking. The discovery of functional dynamical properties of the H3-H4 tetramer in the nucleosomes encourages us to further characterize the H2A and H2B histones in the histone octamer core of the nucleosome, which will provide missing information on the functional dynamics of nucleosomes.

In the present work, we implemented SSNMR to investigate the structure and dynamics of the H2B protein in well-hydrated NCPs precipitated from the solution. The NCP sample is highly hydrated with a concentration of about 370 mg/ml that corresponds to physiological conditions and is within the range of chromatin concentration in vivo (50–500 mg/mL)^41,42^. Multidimensional ^13^C and ^15^N assignments were obtained for all of the R30-A121 residues that are observed in the dipolar-based experiments. The derived secondary structure of the globular domain of H2B in the well-hydrated nucleosome pellet is consistent with the XRD structure of the crystallized NCP. The CANCO profile suggests semi-quantified dynamics of the H2B core at the millisecond-microsecond timescale, inferring that one of the dynamic regions is close to the H4 dynamic domains and another cluster is in the vicinity of DNA. The results suggest that the dynamic networks also extend to H2B in the NCP, which can be potentially critical in modulating DNA activities.

## Results

### H2B R30-A121 were observed in the dipolar-based SSNMR experiments and nearly complete ^13^C and ^15^N resonance assignments were obtained

**Fig. 1:**
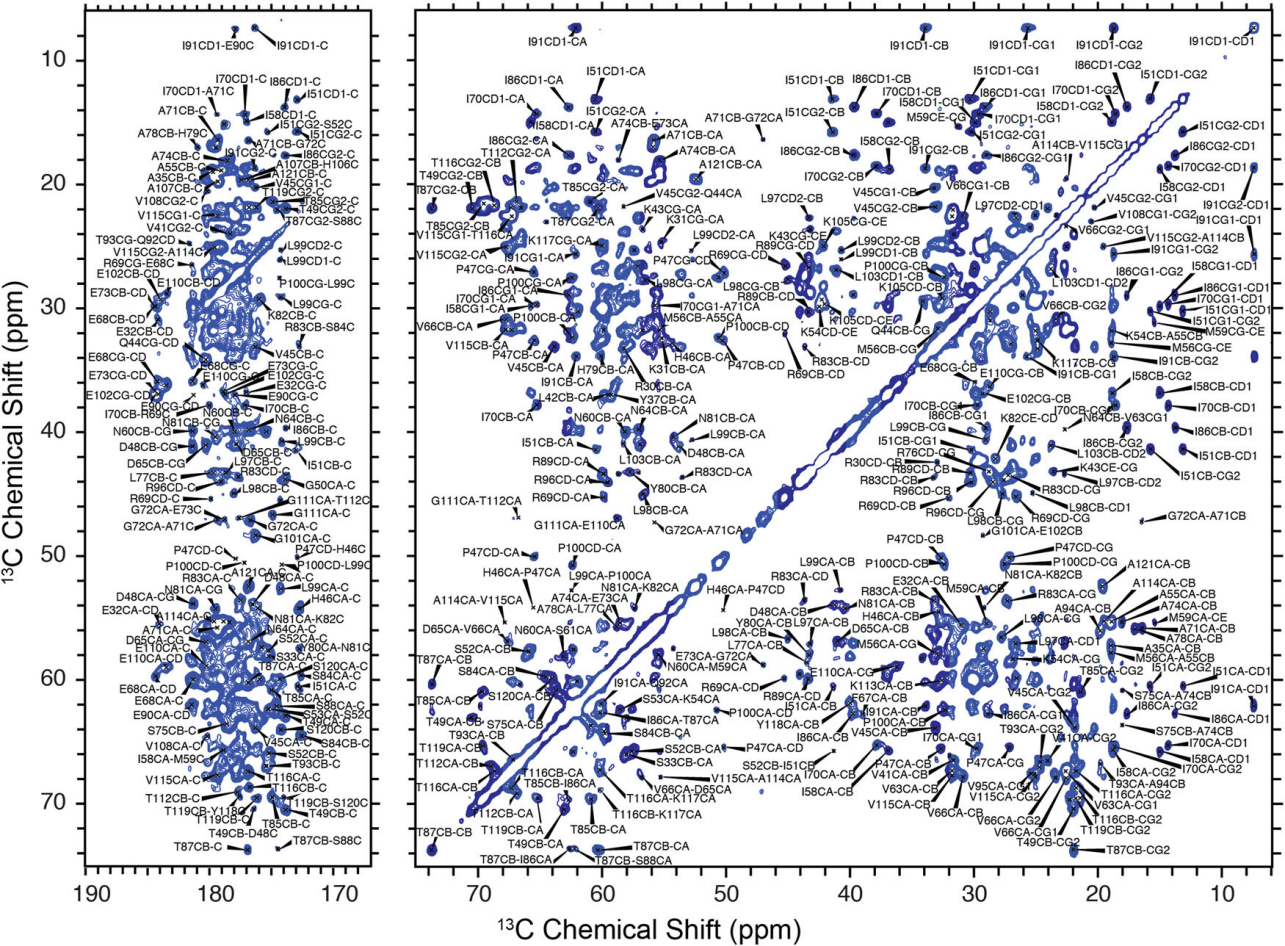
High-quality of the obtained SSNMR spectra allows the resonance assignments for H2B in the NCP sample. 2D ^13^C-^13^C DARR spectrum of H2B in the Mg^2+^ precipitated NCP. The representative partial peak assignments are labeled. 100 ms DARR mixing time was used in the data collection.

A NCP sample was reconstituted from the 145 bp Widom 601 DNA^43^ and the HO containing uniformly ^13^C, ^15^N labeled H2B. The NCP sample was pelleted down with 20 mM Mg^2+^, resulting in a maximum degree of precipitation and columnar hexagonal stacking structure^44^. The water content of the SSNMR sample was 67%, which was determined using the integrals of H_2_O signal and the protein signal in a ^1^H SSNMR spectrum, corresponding to a NCP concentration of 370 mg/ml that is within the range of physiological chromatin concentration^41,42^. The quality of the NCP sample was first assessed by one-dimensional (1D) ^1^H-^13^C CP and two-dimensional (2D) ^13^C-^13^C dipolar-assisted rotational resonance (DARR)^45^ experiments. High resolution of the SSNMR data was obtained as represented by the spectrum shown in Fig. 1, which has similar quality to those in our recent studies of the NCPs harboring uniformly labeled H3 or H4^22,46^. 2D DARR, NCA, NCO, NcaCX, dipolar recoupling enhanced by amplitude modulation (DREAM)^47^, three-dimensional (3D) NCACX, NCOCX and CANCO were collected to first conduct the chemical shift assignments for residues in the globular domain of H2B in the NCP. The high quality of the SSNMR spectra allows us to conduct backbone sequential walk for the H2B as shown in Fig. S1. Overall, nearly complete ^13^C, ^15^N resonance assignments for R30-A121 are obtained using the dipolar-based SSNMR data. The other residues in the H2B N-tails are absent in those dipolar-based SSNMR spectra due to their high flexibility, which can be detected in the J-based SSNMR INEPT experiments (Fig. S2). The schematic plot of the assigned spins is displayed in Fig. S3, and the assignments are deposited in the BioMagResBank database (accession number 51820). This work fills the gap of resonance assignments of histone proteins in nucleosomes, together with our previous characterization of H3^46^ and H4^22^, and another work of H2A by Xiang and coworkers^34^, which together provides SSNMR resonance assignments for all four histone proteins in the nucleosome core particle. It needs to be pointed out that the obtained assignments of H2B are for *Xenopus laevis* histone in this work, those of H3^46^ and H4^22^ are for *Homo sapiens* histones, and those of H2A are for *Drosophila melanogaster* in the previous study^34^. Given the high levels of the core histone conservation across the species, the obtained chemical shift assignments for the four histones allow us to access molecular information by NMR in most scenarios. The availability of resonance assignments for histone proteins in NCP enables the use of solid-state and solution-state NMR to investigate the conformations of histone proteins in nucleosomes and nucleosome–protein complexes, and provides signature peaks to extract amino acid site-specific dynamics.

### Secondary structure of H2B derived from the solid-state NMR resonance assignments is consistent with that of the NCP X-ray crystal structure

**Fig. 2:**
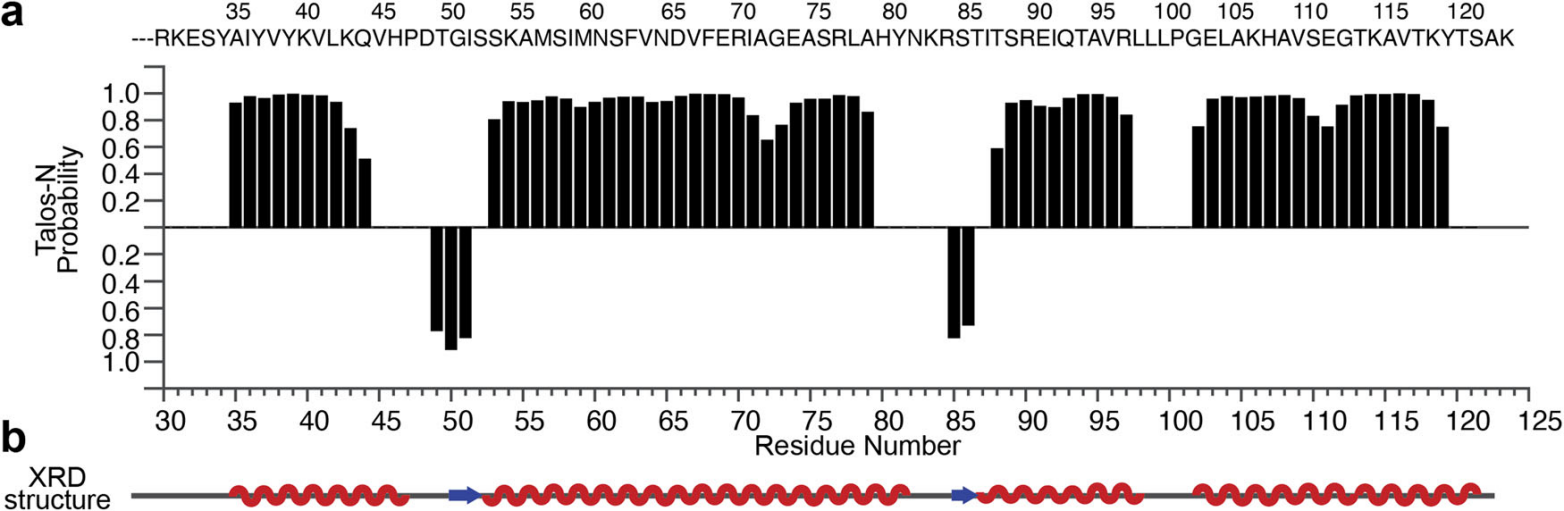
The secondary structure of H2B derived from the obtained SSNMR chemical shifts agrees well with the XRD structure. **a** TALOS-N prediction for H2B in NCP, the primary sequence of H2B R30-K122 is shown on the top. **b** Schematic illustration of the secondary structure of H2B determined by XRD method (PDB: 3lz0^4^).

The secondary structure of H2B in this NCP sample is readily obtained from the chemical shift assignments. Here, we used TALOS-N^48^ to predict the secondary structure of H2B in the well-hydrated NCP pellet sample and the result is shown in Fig. 2. Four helix domains linked by three loops are observed for amino acid sites R30-A121. In addition, short beta-sheet structures exist in the L1 and L3 loops. Those derived secondary structures agree perfectly with XRD data^1,4^. Residues A1-T29 and K122 are absent in the dipolar-based solid-state NMR spectra, which suggests that they are highly dynamic in comparison with the globular domain of H2B, which is consistent with the random coil structures. Overall, the structure information obtained by our current SSNMR measurement for the H2B in this well-hydrated NCP sample is consistent with the XRD structures.

### Amino-acid site-specific microsecond-nanosecond dynamics of H2B revealed a compact HO core

**Fig. 3:**
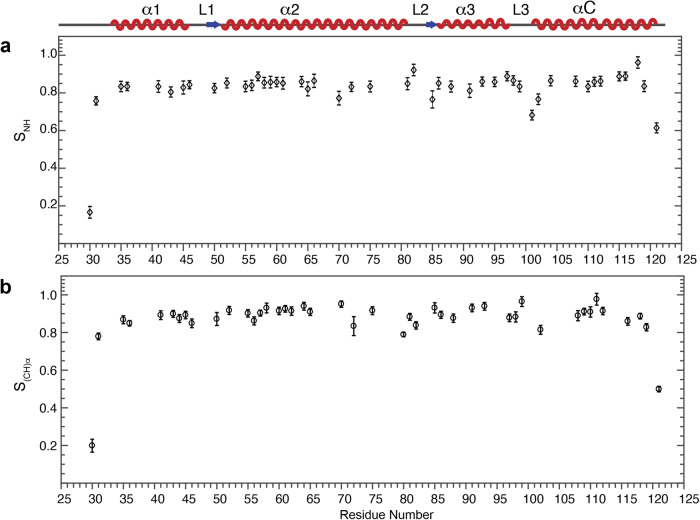
Microsecond-nanosecond dynamics reveal the compact packing of histone core regions in NCP with a few flexible regions. **a** Dipolar order parameters S_NH_ and, **b** S_(CH)α_ determined for H2B in the NCP. The schematic illustration of H2B secondary structure is shown on the top. The experimental and simulated line shapes for all residues are presented in Figs. S4 and S5. Error bars are calculated 95% confidence intervals.

The complete resonance assignments of the globular domain of H2B enable the extraction of the amino-acid site-specific dipolar coupling strengths from 3D dipolar-chemical-shift correlation (DIPSHIFT)^49^ experiments, of which one of the dimensions is the dipolar dephasing trajectory and the other two dimensions are NCA plane for resolving individual amino acid sites. The motionally averaged ^1^H-^13^C and ^1^H-^15^N dipolar coupling constants were calculated by fitting the experimental dipolar lineshapes with simulated data. The calculated order parameters for residues between R30-A121 that can be resolved in the NCA spectrum are presented in Fig. 3. Order parameter values of the backbone (CH)α and NH vectors fall in the range of 0.8-1.0 and fluctuate narrowly between different residues for the sites in the four helical regions and the loops L1 and L2. Residues R30 and K31 belong to the N-terminal tail region adjacent to the α1 helix, thus the lower smaller order parameters are consistent with the highly flexible structure of the N-terminal tail. Similarly, residues T119 and A121 possess reduced order parameters, reflecting higher flexibility at the microsecond-nanosecond timescale of T119-K122 that belong to the short C-terminal region and the adjacent partial αC helix. Smaller values of the order parameters are also detected for residues in the L3 loop, showing that this domain exhibits enhanced mobility in the microsecond-nanosecond timescale. This indicates the increased plasticity of the center of the histone core in the NCP, where the packing density is lower. In summary, the narrow range of the order parameters determined for the backbone of the majority of the globular domain of H2B in the NCP suggests a rigid and tight structural packing of the histone folds, which provides a stable platform for wrapped DNA activities. In addition, the N-terminal and C-terminal regions, as expected, exhibit enhanced mobility at the microsecond-nanosecond timescale, and the L3 region displays higher flexibility in comparison with the majority of the globular domain.

### The CANCO peak profile suggests the extension of dynamic networks to the H2B in NCP

**Fig. 4:**
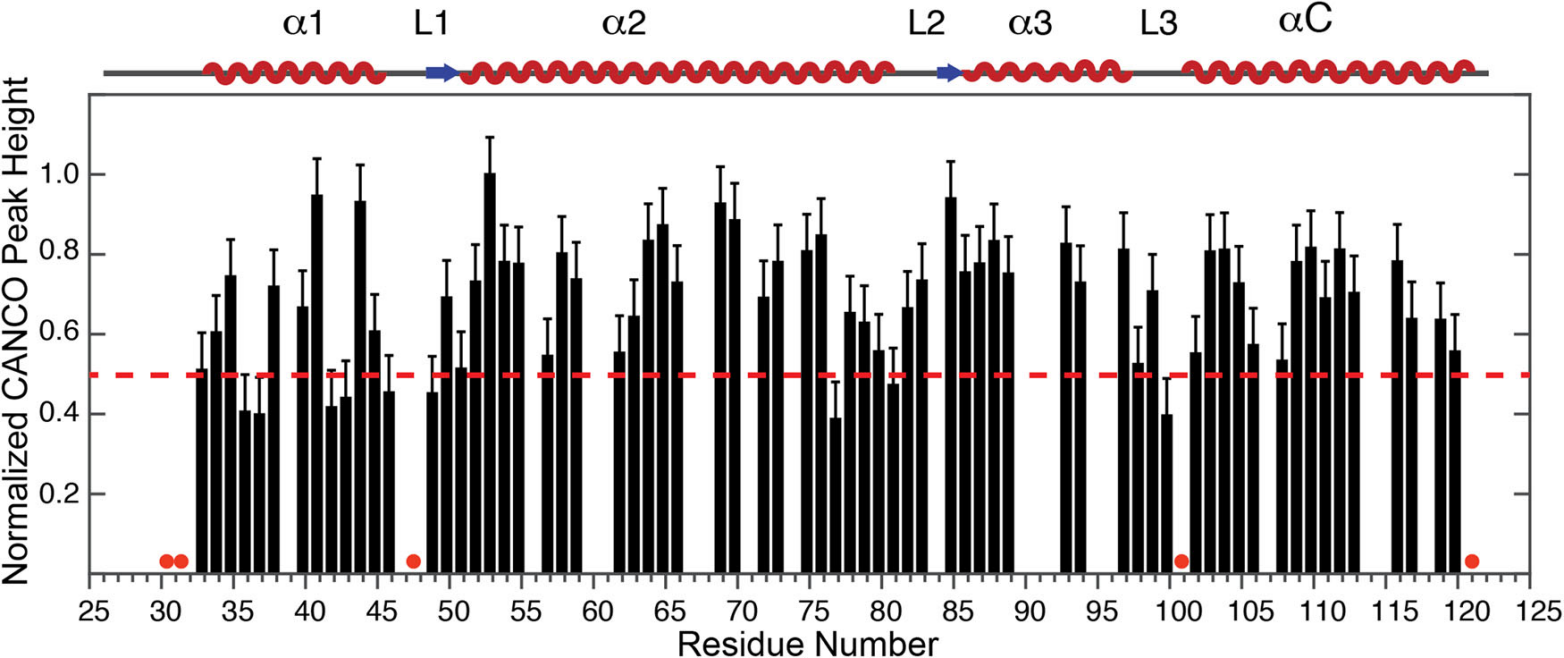
CANCO profile shows considerable peak intensity differences among different residues of H2B globular domain in the NCP. The CANCO peak heights are plotted as a function of residue number. The peak heights are normalized to the highest value in the dataset. Error bars are calculated from the RMSD values of the CANCO spectral noise. Red dots indicates residues in the H2B globular domain, of which the inter-residue CANCO peaks are absent in the spectrum. A red dashed line corresponding to a y-axis value of 0.5 is drawn to guide visualization.

**Fig. 5:**
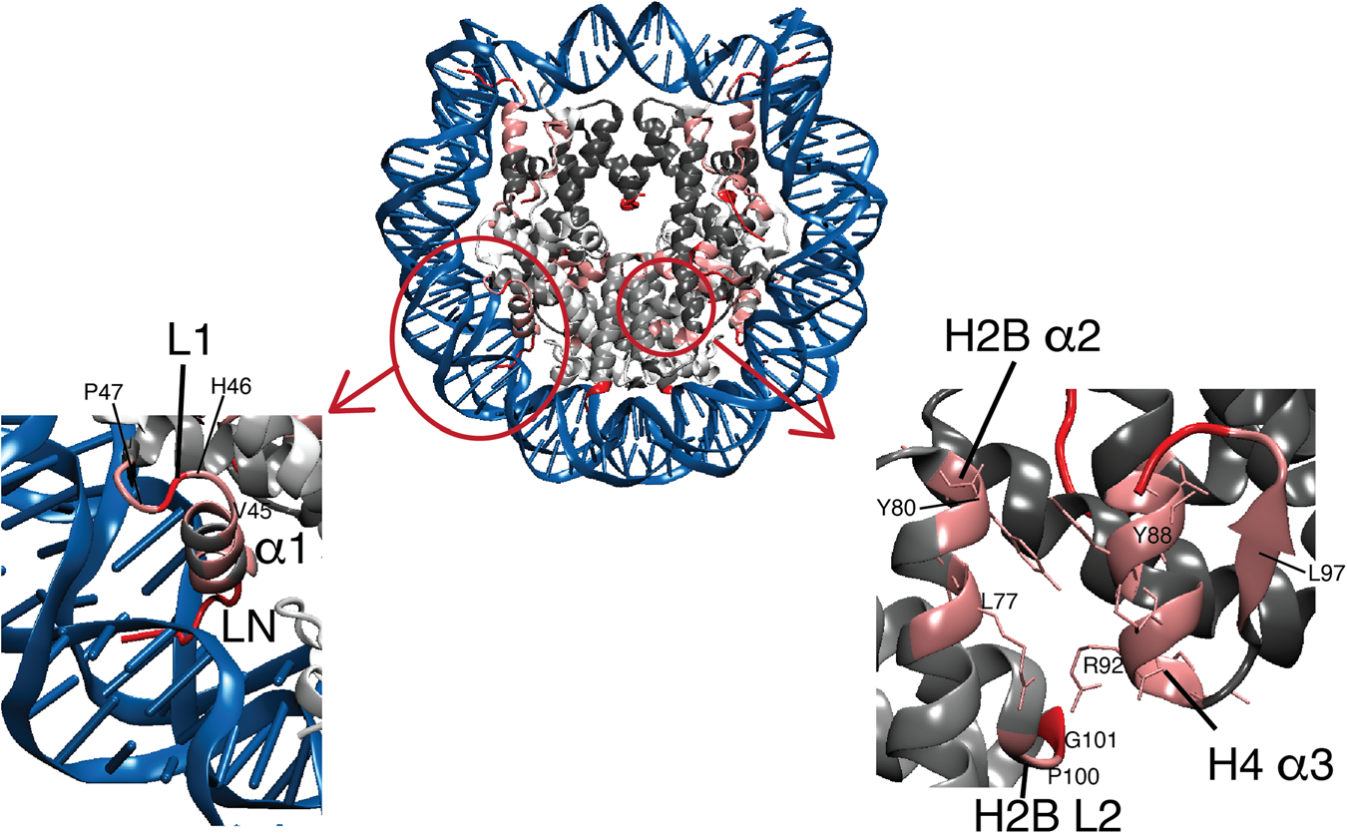
H2B residues exhibiting higher millisecond-microsecond dynamics form clusters in the NCP. The dynamical residues corresponding to those showing zero or weaker 3D CANCO peak signals (normalized intensity < 0.5) are highlighted in red and pink, respectively, in the NCP structure. Zoom-in pictures of two regions containing clustered dynamical residues are shown at the bottom. In the bottom right pannel, the sidechains of dynamics residues in the H2B α2 and H4 α3 are also shown to illustration their spacial contacts. A few residues are marked to help identifying the relative locations of different regions.

Our previous studies of the H3 and H4 proteins in the NCP demonstrated the existence of dynamic networks in the histone core, formed by amino acid residues that possess higher mobility^39^. Those networks connect the histone core regions with afar DNA sites and can play dominant roles in regulating DNA activities in a manner analogous to allosteric regulation. In order to reveal a more complete picture of the dynamics at the millisecond-microsecond timescale in the histone core, here, we semi-quantitatively determine the mobility for the H2B amino acid residues in the NCP. Fig. 4 displays the normalized CANCO intensity peaks for residues of H2B that can be well resolved in the 3D spectra. The dipolar-based CANCO experiment contains two magnetization transfer steps through C-N dipolar coupling interactions. Consequently, the spectral peak intensities mostly reflect the mobility at the millisecond-microsecond timescale. The CANCO intensity profile indicates that some regions possess relatively higher mobility. Among residues that are detected in the dipolar-based experiments, the K31-E32, V45-H46, and P100-G101 regions are absent in the CANCO spectrum, illustrating their significantly higher mobility at the millisecond-microsecond timescale. It is noted that R30-K31 and S120-A121 are also absent in the CANCO spectra, which can also be attributed to fast motions at the sub-microsecond as indicated by the smaller order parameters obtained from the dipolar coupling measurements. In addition, the rest of the globular domain shows considerable peak intensity differences between different regions as indicated in Fig. 4. A recent 15-microsecond MD simulation study observed microsecond motion events occurring for both histone tails and histone core regions, which governs DNA unwrapping^23^. Therefore, besides the fast nanosecond-microsecond motions, histone N-terminal tails are likely involved in motions at the millisecond-microsecond timescale. We next summarize the residues of H2B that possess relatively higher dynamics and explore their connections with the dynamic network previously observed for the H3 and H4 histones in NCP. In Fig. 5, the regions highlighted in red and pink correspond to residues that possess the highest (absent in CANCO) flexibility and relatively higher (displaying smaller CANCO intensity) millisecond-microsecond dynamics. The regions in H2B that exhibit higher mobility primarily include the N-terminal region, and the partial α1, L1, α2, and L3 regions as well as the C-terminal end. As illustrated in Fig. 5, the N-terminal region, the partial α1 and L1 regions, cluster together and are in close contact with DNA. Interestingly, the residues of the partial α2 region with higher flexibility are in the vicinity of the H4 α3 region that was determined to exhibit enhanced dynamics in our previous work^39^. This clustering of regions with higher mobility suggests that the dynamic networks of the H3/H4 tetramer also extend to the H2B/H2A dimer although there is no quantified dynamics data determined for H2A yet. Two additional short regions possessing higher mobility are the partial L3 region and the C-terminal end, of which the former locates at the center of the histone core with low packing density. The higher dynamics of those regions likely correspond to their structural packing features.

## Discussion

Chromatin is a highly dynamic DNA-protein complex with structure and dynamics regulated by various factors. The dynamics of chromatin at both the microscale and the mesoscale levels are critical to its biological functions. The previous studies from us and other groups suggested a significant functional relevance of millisecond-microsecond timescales in nucleosomes and the existence of dynamic networks that potentially enable coupling between the histone core regions afar from DNA sites, which could potentially drive the transmission of signals generated by changes such as epigenetic modifications. In this work, we conducted a SSNMR study to explore the structure and internal dynamics of the H2B histone in NCP with a close to physiological concentration. The obtained ^13^C, ^15^N chemical shift assignments of H2B make a complete database for chemical shift assignments available for all four histones in nucleosomes. The H2B in this well-hydrated NCP pellet folds into the same structure as previously determined by XRD studies. Order parameters derived from the dipolar coupling constant measurements and CANCO peak intensity profile provide information about the dynamics at the microsecond-nanosecond and millisecond-microsecond timescales for H2B in NCP in a site-specific manner. The majority of the globular domains possess similar microsecond-nanosecond mobility, inferring a stable and compact histone core region. Several regions were observed to show relatively higher millisecond-microsecond dynamics in the H2B core, one of which is in close contact with DNA and the other clusters together with one of the H3-H4 dynamic networks. This illustrates that dynamics networks also exist in the H2A-H2B dimers and they potentially interact with those in the H3-H4 tetramer and with DNA. A recent study has reported the predicted order parameters using the random coil index (RCI) method for H2A in a NCP^34^, however, the experimental determination of H2A dynamics is still lacking. A study on H2A dynamics at different timescales is awaiting future studies to elucidate the full picture of the dynamic networks in the nucleosome. Overall, the current work provides an invaluable characterization of the internal dynamics of histones in nucleosomes and key fingerprint spectra for future NMR studies of chromatin.

The last piece of information, which is the dynamics of H2A, is still lacking for a more complete understanding of the dynamics of histone core in nucleosomes. In addition, the dynamics was obtained in a semi-quantitative manner and only for amino acid backbone residues. Future experiments employing other SSNMR techniques^50,51^ or in combination with MD simulations can be conducted to elucidate the motions of amino acid sidechains and DNA at a higher spatial and temporal resolution, although such experiments are challenging for these large-size complexes. Furthermore, exploring the functional relevance of the dynamic networks observed for the nucleosome requires more parallel biological studies similar as to our recent work^38^.

## Methods

### Preparation of nucleosome core particle

Histone proteins were overexpressed by *Escherichia coli* BL 21 (DE3) pLysS strain. The cell culture were grown in 2x YT medium at 37 °C till OD_600_ reaching 0.5, and then was induced with 0.4 mM IPTG. Cells were harvested after 3-hour incubation at 37 °C. The inclusion bodies were extracted and dissolved in unfolding buffer containing 7 M guanidinium HCl, 20 mM sodium acetate (pH 5.2), 10 mM DTT (or 20 mM DTT used for H3 preparation). The crude histone solution was subsequently purified using a HiPrep 26/60 Sephacryl S-200 HR column (GE Healthcare) and the elution fractions were dialyzed against Milli-Q water containing 5 mM beta-mercaptoethanol. For histone fractions containing DNA contamination, a second step of purification by ion-exchange chromatography using a RESOURCE S column (Cytiva). The purified proteins were lyophilized and stored at −80 °C till further usage. To obtain the isotopically ^13^C, ^15^N labeled *Xenopus laevis* H2B, cells were first grown at 2x YT media till the OD reached 0.5, and then were spun down at room temperature. The collected cells were re-suspended in M9 medium containing 2 g/L ^13^C-glucose and 1 g/L ^15^N-NH_4_Cl and were incubated at 37 °C for one hour before induction with 0.4 mM IPTG.

To prepare the histone octamer (HO), the lyophilized ^13^C, ^15^N labeled *Xenopus laevis* H2B and natural abundant *Homo sapiens* H2A, H3 and H4 were dissolved in unfolding buffer containing 7 M guanidinium HCl, 10 mM Tris-HCl (pH 7.5) and 10 mM DTT (20 mM DTT for H3). The solution of four different histones were mixed at equimolar ratio. The mixture was dialyzed against the refolding buffer containing 2 M NaCl, 10 mM Tris-HCl (pH 7.5), 1 mM sodium-EDTA, 5 mM beta-mecaptoethanol at 4 °C. The obtained HO solution was purified by FPLC gel filtration using a HiLoad 16/60 Superdex column 200 pg (GE Healthcare). The fractions were evaluated by 18% SDS gel (Fig. S6) and the pure HO were combined for the subsequent reconstitution.

The 145 bp Widom 601 DNA was obtained by amplification of a pUC19 plasmid harboring eight copies of the DNA fragments with DH5α strain. The cells were grown in TB medium and harvested after 21-h growth at 37 °C. The plasmids were extracted using the alkaline lysis method and digested by EcoRV-HF (NEB). The 145 bp 601 DNA were separated from the backbone vector using polyethylene glycol fractionation.

NCP was reconstituted by the conventional salt gradient dialysis method^52^. The DNA and HO containing uniformly ^13^C, ^15^N labeled H2B were mixed in a starting buffer containing 2 M KCl, 1 mM EDTA, and 1 mM DTT. The mixture was transferred into a 3 kDa dialysis bag and placed in the starting buffer that is constantly removed and replaced by the buffer free of KCl using a peristaltic pump for 20-24 h at a flow rate of 0.5 ml/min at room temperature. Subsequently, the mixture was dialyzed against the buffer without KCl for another 2-3 h to ensure the KCl concentration reaching below 0.2 M. The reconstituted product was evaluated by 6% native PAGE and 18% SDS-PAGE (Fig. S6).

The reconstituted NCP that contains uniformly ^13^C, ^15^N labeled H2B was concentrated to 3 mg/ml in buffer containing 20 mM Tris pH 7.5, 1 mM EDTA, 1 mM DTT, <0.2 M KCl (from salt gradient dialysis) and transferred to a SSNMR sample loading device (Giotto tech), 1 M MgCl_2_ was added into the NCP solution to a final concentration of 20 mM Mg^2+^ to induce maximum NCP precipitation. The sample was centrifuged at 100,000 xg at 4 °C for 2-3 h to spin the NCP pellet down to a 3.2 mm SSNMR rotor.

### Solid-state NMR experiments

All of the SSNMR experiments were performed on an 18.8 T Bruker Advance III HD spectrometer equipped with a 3.2 mm EFree probe. The magic angle spinning (MAS) rate was 15151 Hz and the temperature was regulated with a BCU-II unit to maintain the sample temperature at 12 °C, which was calibrated using ethylene glycol^53^. 2D CC DARR with different mixing times, 2D CC dipolar recoupling enhanced by DREAM scheme, 2D NcaCX, 3D NCACX, NCOCX and CANCO were performed to obtain ^13^C, ^15^N assignments. 3D DIPSHIFT experiments using R12_1_^4,54,55^ symmetry sequences on ^1^H channel in a constant-time manner were performed to extract ^1^H-^13^C and ^1^H-^15^N dipolar lineshapes. The detailed experimental parameters were listed in Table S1 in the Supplementary Information. Data were processed using NMRPipe^56^ and analyzed with SPARKY (T. D. Goddard and D. G. Kneller, University of California, San Francisco). The ^13^C chemical shifts were referenced using adamantine as the external standard with the downfield carbon signal set to 40.48 ppm^57^, and the ^15^N chemical shift reference was indirectly calculated. The ^1^H-^13^C and ^1^H-^15^N dipolar recoupling trajectories were extracted in NMRPipe and fitted using SIMPSON^58^.

### Statistics and reproducibility

3D NCACX, NCOCX, CANCO experiments were collected 10-16 times and were co-added together for analysis. 3D DIPSHIFT experiments were performed 13-14 times and co-added together for analysis. The deviation of error bars for order parameters displayed in Fig. 3 and those represented in Fig. 4 are described in the corresponding figure captions.

### Reporting summary

Further information on research design is available in the Nature Portfolio Reporting Summary linked to this article.

## Supplementary information

Supplementary Information

Description of Additional Supplementary Files

Supplementary Data

Reporting Summary

## Data Availability

The full chemical shift assignments obtained in this work are available in BioMagResBank database under the accession number 51820. Data for making Figs. 2, 3 and 4 are available as Supplementary Data and any remaining information can be obtained from corresponding authors upon reasonable request.
