# Supplementary material for: Structural and dynamical investigation of histone H2B in well-hydrated nucleosome core particles by solid-state NMR
Source: Commun Biol. 2023 Jun 24;6:672. doi: 10.1038/s42003-023-05050-3 (PMC10290710; doi:10.1038/s42003-023-05050-3)
Supplement: Supplementary file 1 — Supplementary Information [file 42003_2023_5050_MOESM1_ESM.pdf]

Structural and Dynamical Investigation of Histone H2B in Well-hydrated Nucleosome Core Particles by Solid-state NMR

Xiangyan Shi[1]*, Bhuvaneswari Kannaian[2], Chinmayi Prasanna[2,4], Aghil Soman[2], Lars Nordenskiöld[2,3]*

1. Department of Biology, Shenzhen MSU-BIT University, Shenzhen, Guangdong Province, China.

2. School of Biological Sciences, Nanyang Technological University,Singapore.

3. NTU Institute of Structural Biology, Nanyang Technological University, Singapore.

4. Present address: Department of Physiology and Biophysics, Case Western Reserve University, OH, USA.

Email: xyshi@smbu.edu.cn (XS), LarsNor@ntu.edu.sg (LN)

Table S1 SSNMR parameters of experiments performed for the Widom '601' NCP containing uniformly ^{13}C, ^{15}N labeled H2B.

Experiment	CC	CC	CC	CC	NCA	NCO
**Field (T)**	18.8	18.8	18.8	18.8	18.8	18.8
**MAS rate (kHz)**	15.151	15.151	15.151	15.151	15.151	15.151
**transfer 1**	HC CP	HC CP	HC CP	HC CP	HN CP	HN CP
rf field (kHz), ^{1}H<br>shape	72.9<br>ramp	72.9<br>ramp	73.1<br>ramp	72.9<br>ramp	51.6<br>ramp	51.6<br>ramp
rf field (kHz), $^{15}N/^{13}C$	53.0	53.0	53.0	53.0	37.9	37.9
transfer time (ms)	1.0	1.0	1.2	1.2	1.0	1.0
carrier (ppm)<br>^{13}C,^{15}N	103.0<br>–	103.0<br>–	103.0<br>–	103.0<br>–	–<br>115.4	–<br>115.4
**transfer 2**	DARR	DARR	DREAM	DREAM	NCA SPECIFIC	NCO SPECIFIC
rf field (kHz), ^{13}C<br>shape	–	–	9.9<br>tangent	9.9<br>tangent	25.5<br>tangent	41.8<br>tangent
rf field (kHz), ^{15}N	–	–	–	–	37.9	22.7
rf field (kHz), ^{1}H cw	13.6	13.6	73.7	73.7	86.0	86.0
transfer time (ms)	20	100	3.0	3.0	3.5	3.5
carrier (ppm)<br>^{13}C,^{15}N	103.0<br>–	103.0<br>–	57.0<br>–	30.0<br>–	58.0<br>115.4	178.0<br>115.4
**transfer 3**	–	–	–	–	–	–
rf field (kHz), ^{13}C	–	–	–	–	–	–
rf field (kHz), ^{15}N	–	–	–	–	–	–
rf field (kHz), ^{1}H	–	–	–	–	–	–
transfer time (ms)	–	–	–	–	–	–
carrier (ppm)<br>^{13}C,^{15}N	–<br>–	–<br>–	–<br>–	–<br>–	–<br>–	–<br>–
**digitalization, F1**	C	C	C	C	N	N
t1 increments	768	768	256	256	192	192
sweep width (kHz)	45453	45453	15151	15151	7575.5	7575.5
acquisition time (ms)	8.4	8.4	8.4	8.4	12.7	12.7
**digitalization, F2**	C	C	C	C	C	C
t2 increments	1536	1536	1280	1280	832	832
sweep width (kHz)	53571.43	53571.43	45454.547	45454.547	29761.904	29761.904
acquisition time (ms)	14.3	14.3	14.1	14.1	14.0	14.0
**digitalization, F3**	–	–	–	–	–	–
t3 increments	–	–	–	–	–	–
sweep width (kHz)	–	–	–	–	–	–
acquisition time (ms)	–	–	–	–	–	–
**^{1}H decoupling**<br>**rf filed (kHz)**<br>**shape**	70.7<br>SPINAL 64	70.7<br>SPINAL 64	70.7<br>SPINAL 64	70.7<br>SPINAL 64	70.7<br>SPINAL 64	70.7<br>SPINAL 64
**pulse delay (s)**	1.5	1.5	1.5	1.5	1.5	1.5

Table S1 (Continued).

Experiment	NcaCX	NCACX	NCOCX	CANCO	NCACX	NCOCX
**Field (T)**	18.8	18.8	18.8	18.8	18.8	18.8
**MAS rate (kHz)**	15.151	15.151	15.151	15.151	15.151	15.151
**transfer 1**	HC CP	HN CP	HN CP	HC CP	HN CP	HN CP
rf field (kHz), ^{1}H	51.8	51.8	51.8	71.3	52.9	52.9
shape	ramp	ramp	ramp	ramp	ramp	ramp
rf field (kHz), $^{15}N/^{13}C$	37.9	37.9	37.9	53.0	37.9	37.9
transfer time	1.0	1.0	1.0	0.8	1.0	1.0
carrier (ppm)	103.0	–	–	55.0	–	–
$^{13}C,^{15}N$	–	115.4	115.4	–	115.4	115.4
**transfer 2**	NCA SPECIFIC	NCA SPECIFIC	NCO SPECIFIC	CAN SPECIFIC	NCA SPECIFIC	NCO SPECIFIC
rf field (kHz), ^{13}C	25.4	25.4	40.9	25.4	24.9	41.4
shape	tangent	tangent	tangent	tangent	tangent	tangent
rf field (kHz), ^{15}N	37.9	37.9	22.7	37.9	37.9	22.7
rf field (kHz), ^{1}H cw	86.0	86.0	86.0	86.0	86.0	86.0
transfer time	3.5	3.5	3.5	3.0	3.5	3.5
carrier (ppm)	55.0	55.0	178.0	55.0	55.0	178.0
$^{13}C,^{15}N$	115.4	115.4	115.4	115.4	115.4	115.4
**transfer 3**	DARR	DARR	DARR	NCO SPECIFIC	DARR	DARR
rf field (kHz), ^{13}C	–	–	–	40.9	–	–
rf field (kHz), ^{15}N	–	–	–	22.7	–	–
rf field (kHz), ^{1}H	13.6	13.6	13.6	86.0	13.6	13.6
transfer time	75	75	75	3.0	20	20
carrier (ppm)	103.0	103.0	103.0	178.0	103.0	103.0
$^{13}C,^{15}N$	–	–	–	115.4	–	–
**digitalization, F1**	N	N	N	CA	N	N
t1 increments	64	48	48	84	48	48
sweep width (kHz)	3030.2	3030.2	3030.2	7575.5	3030.2	3030.2
acquisition time (ms)	10.6	7.9	7.9	5.5	7.9	7.9
**digitalization, F2**	C	CA	CO	N	CA	CO
t2 increments	1280	84	36	48	84	36
sweep width (kHz)	45454.547	7575.864	3030.2	3030.2	7575.864	3030.2
acquisition time (ms)	14.1	5.5	5.9	7.9	5.5	5.9
**digitalization, F3**	–	C	C	CO	C	C
t3 increments	–	1088	1088	1088	1088	1088
sweep width (kHz)	–	45454.547	45454.547	45454.547	45454.547	45454.547
acquisition time (ms)	–	12.0	12.0	11.9	12.0	12.0
**^{1}H decoupling rf filed (kHz) shape**	70.7 SPINAL 64	70.7 SPINAL 64	70.7 SPINAL 64	70.7 SPINAL 64	70.7 SPINAL 64	70.7 SPINAL 64
**pulse delay (s)**	1.5	1.5	1.5	1.5	1.5	1.5

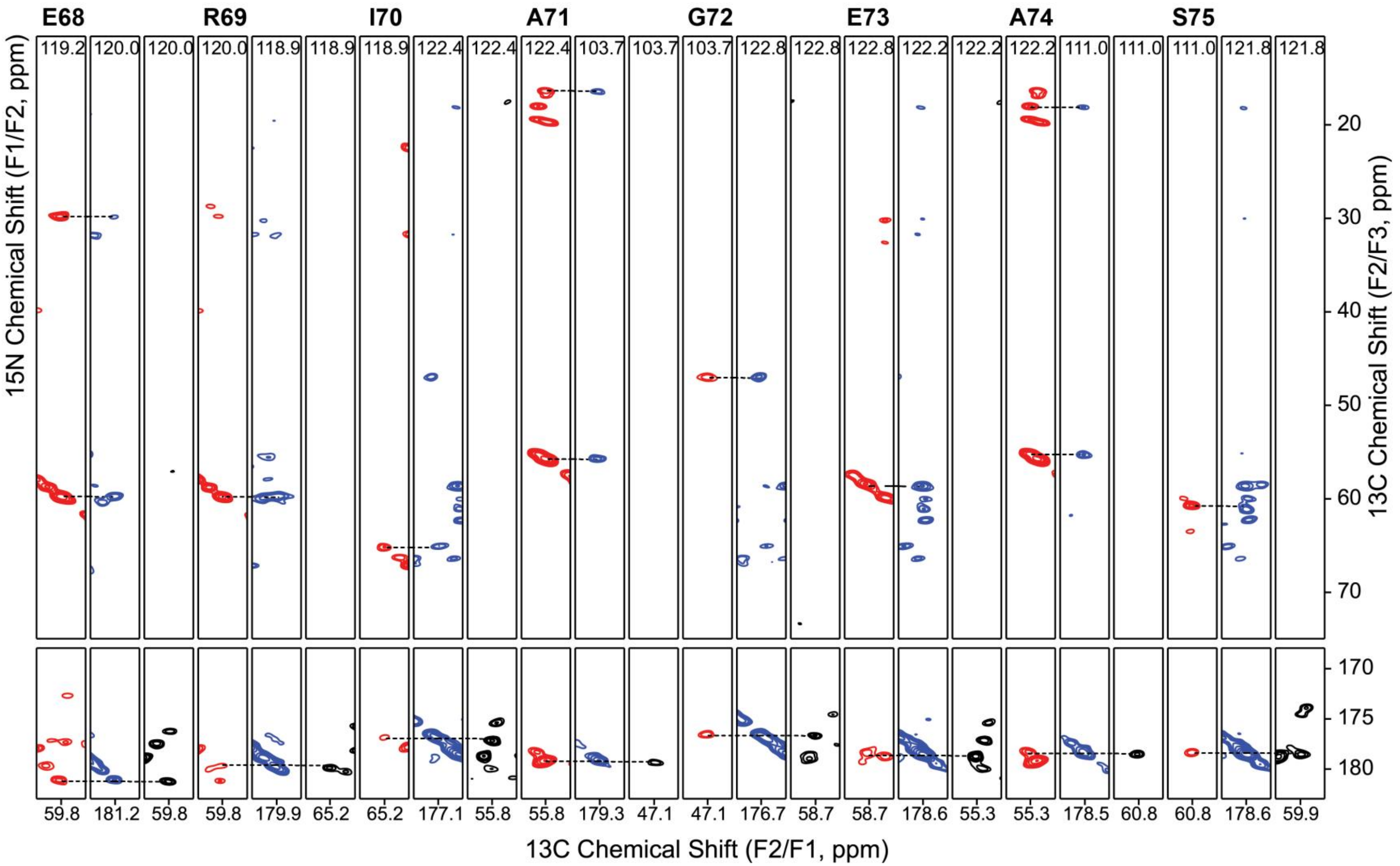

Fig. S1 Representative sequential assignment walk for the E68-I91 fragment in H2B in NCP. Strip plots of 3D NCACX (red), NCOCX (blue) and CANCO (black) spectra used for the sequential assignment walk are shown. The sequential connections are marked by dash lines showing inter- and intra- residue correlations.

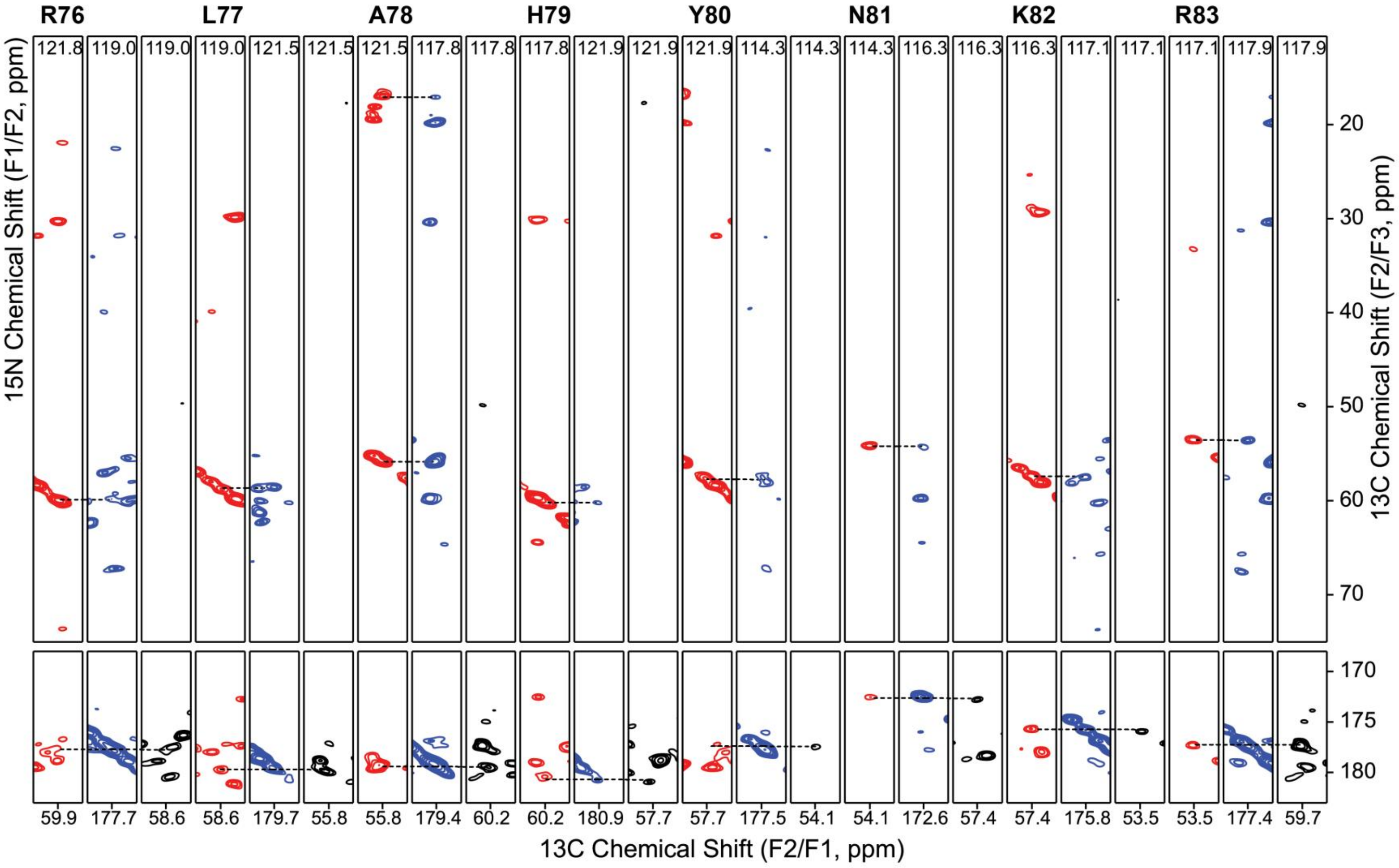

Fig. S1 (Continued).

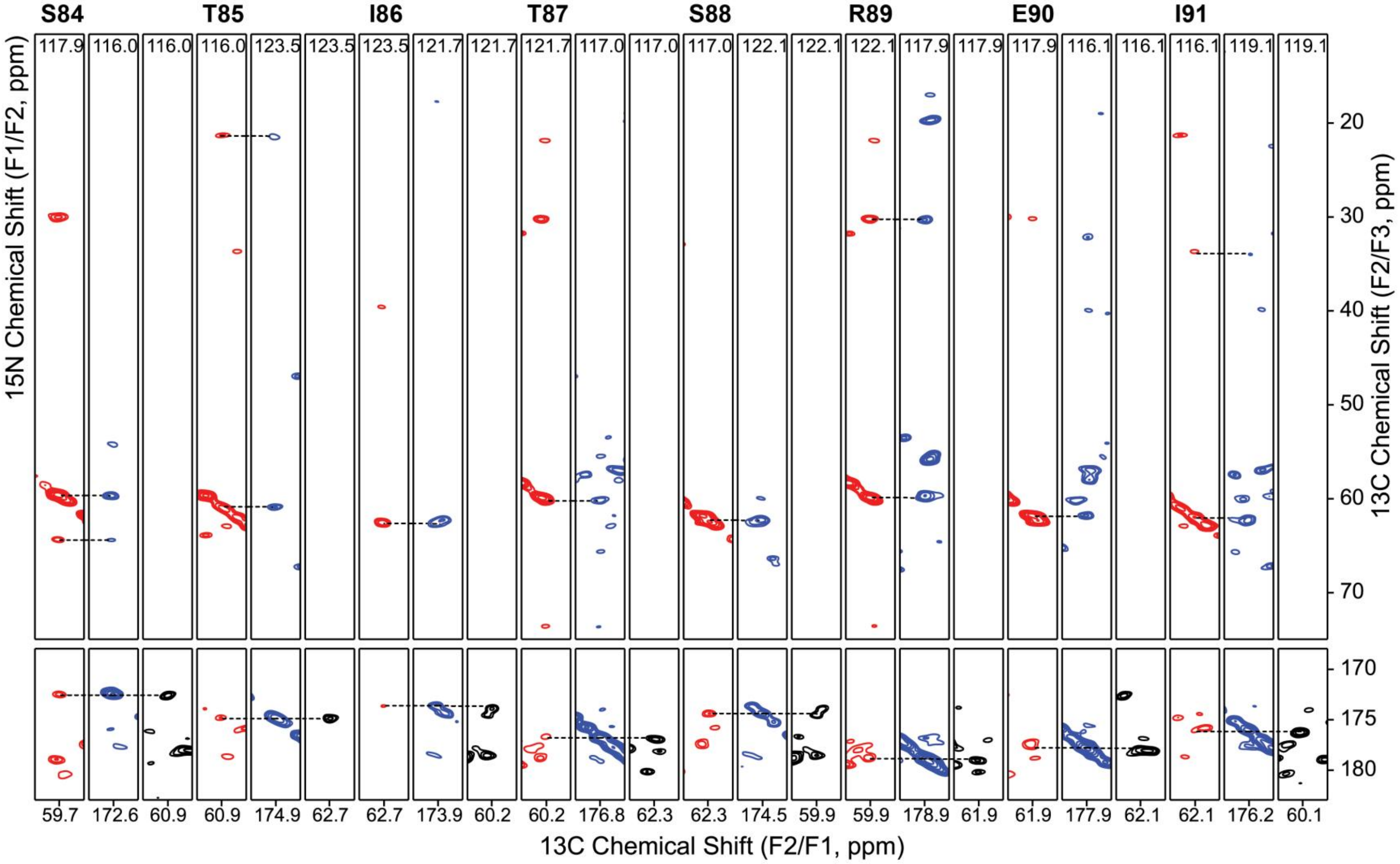

Fig. S1 (Continued).

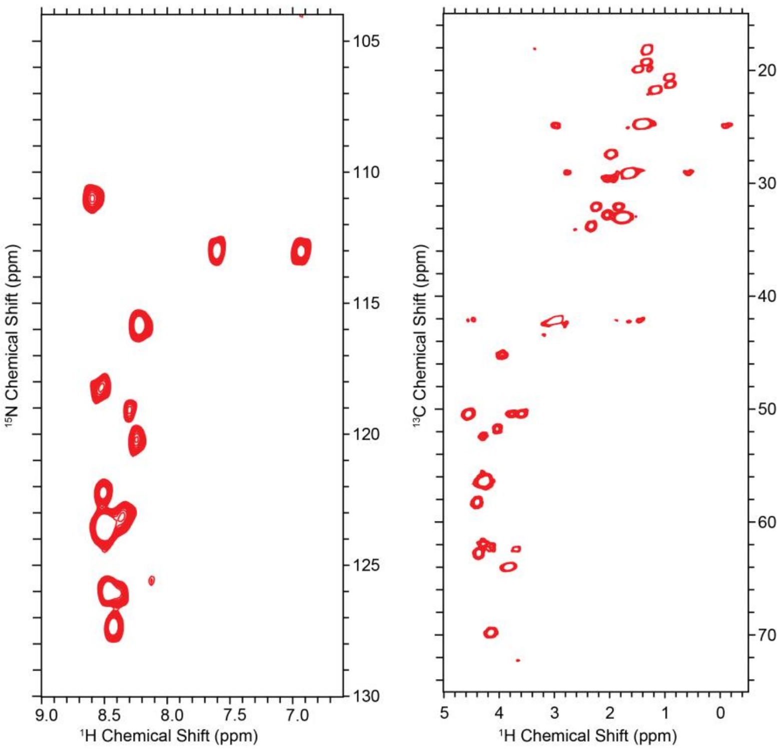

Fig. S2 ^{1}H-^{15}N (left) and ^{1}H-^{13}N (right) *J*-based INEPT correlation SSNMR spectra obtained for the Widom 601 NCP sample containing uniformly ^{13}C, ^{15}N labeled H2B.

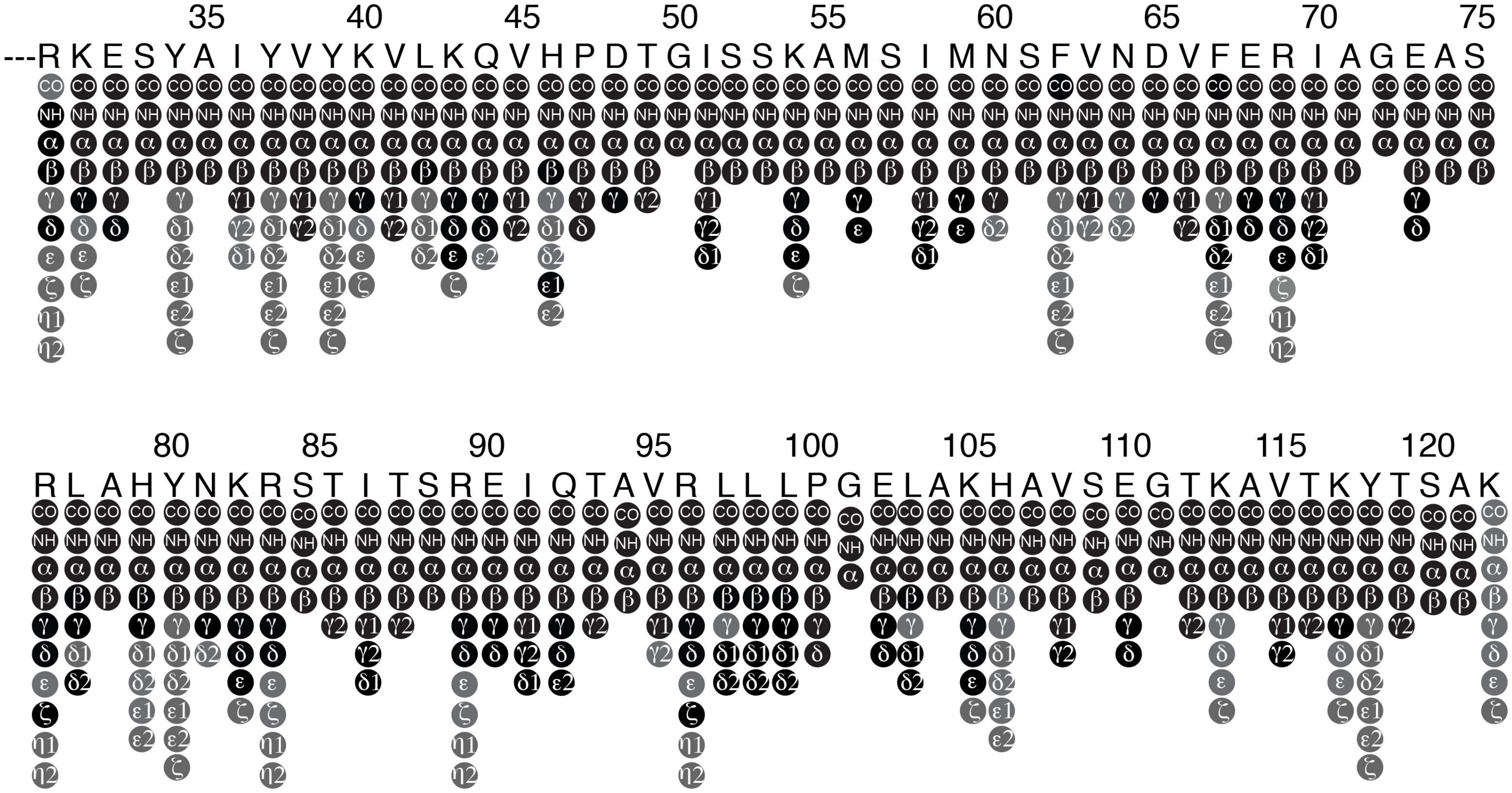

Fig. S3 Sequential assignment graph showing carbon and nitrogen spins of H2B R30–A121. The assigned and unassigned spins are shown in black and grey circles, respectively.

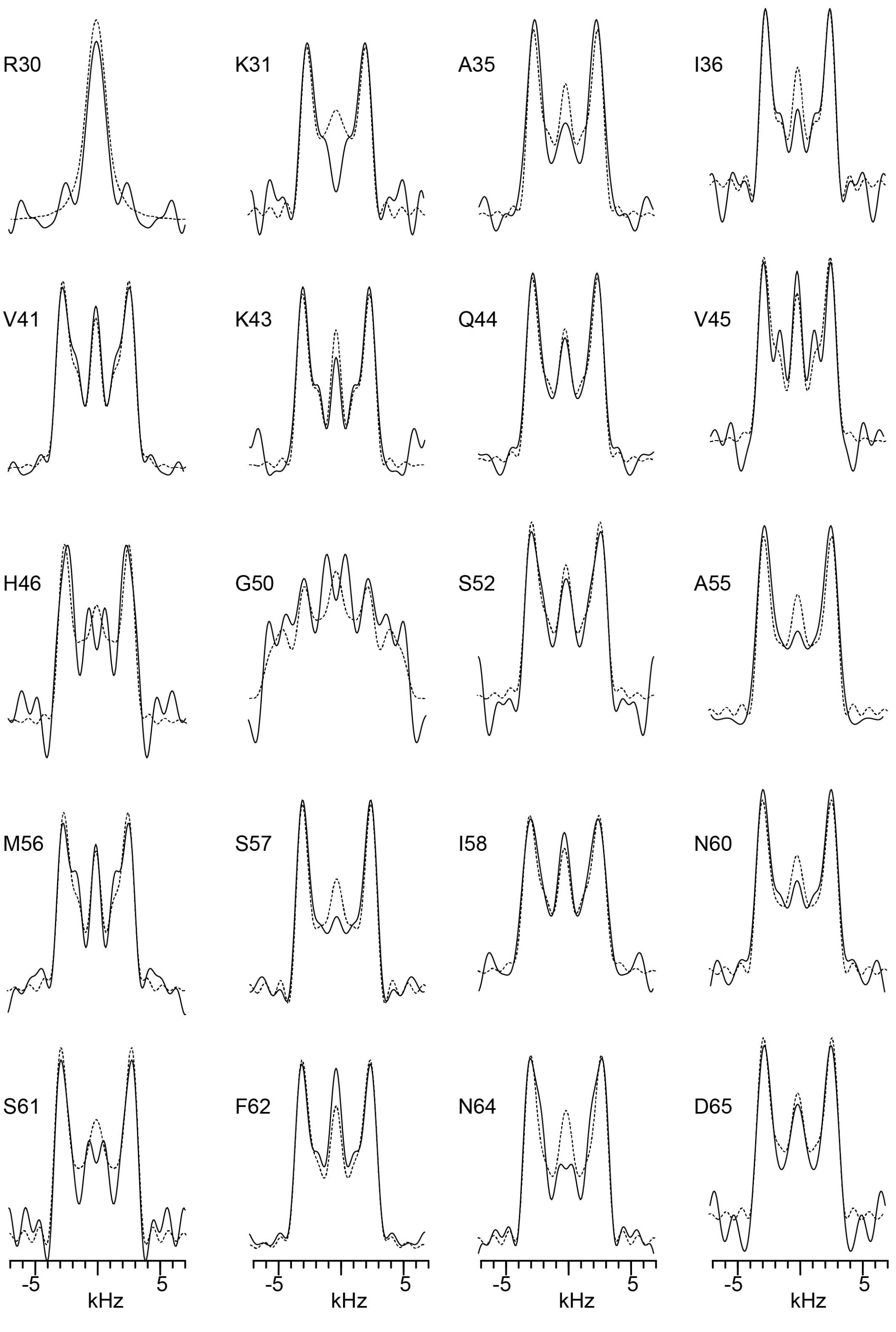

Fig. S4 Site-resolved experimental (solid) and simulated (dotted) ^{1}H-$^{13}C\alpha$ dipolar line shapes for H2B in a Widom 601 NCP. Experimental line shapes are extracted from a 3D DIPSHIFT with $R12_1^4$ symmetry dipolar recoupling pulses. The simulated line shapes were obtained by using SIMPSON .

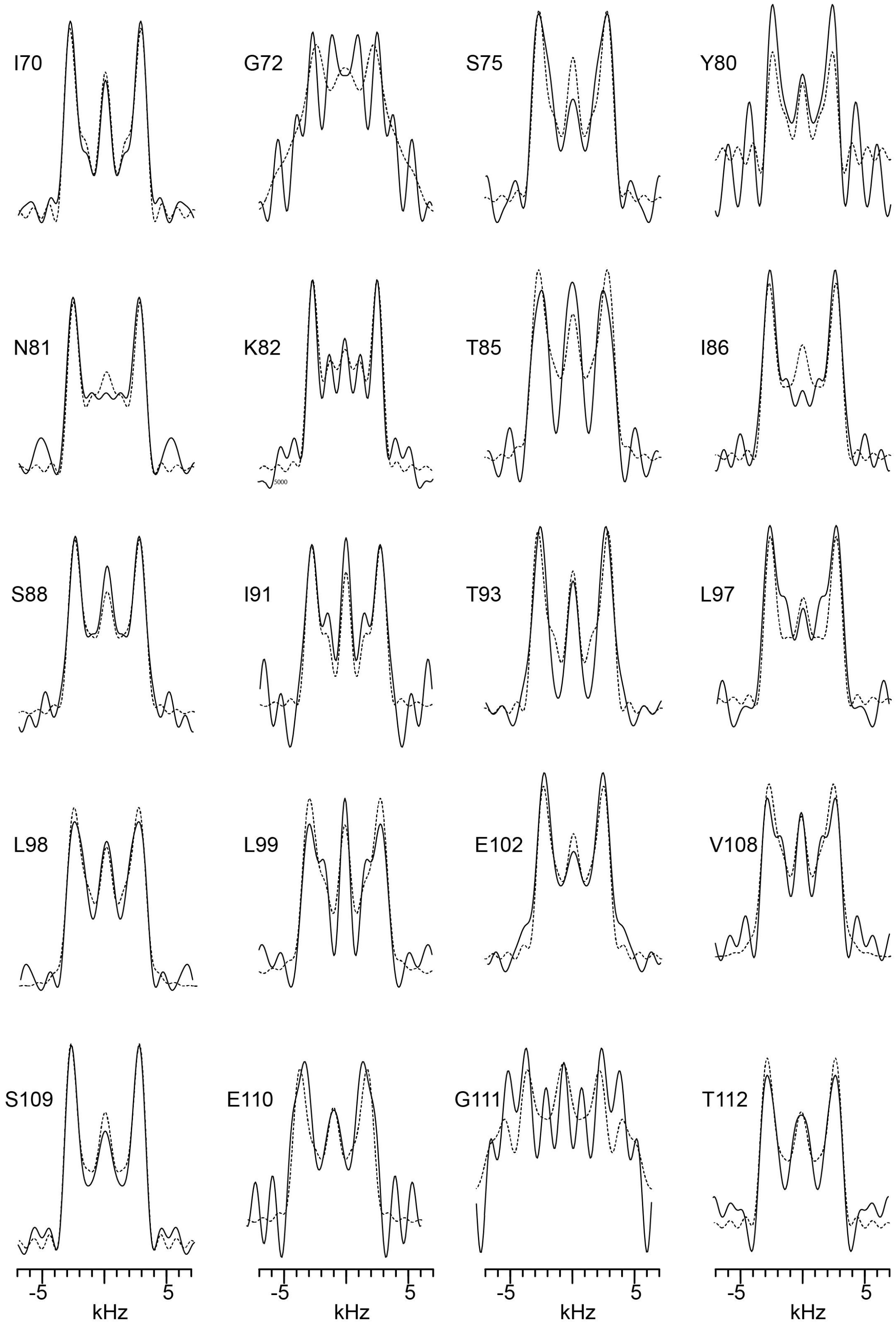

Fig. S4 (Continued)

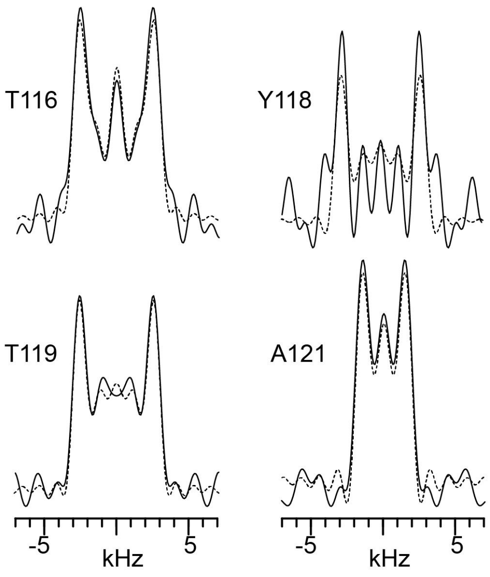

Fig. S4 (Continued)

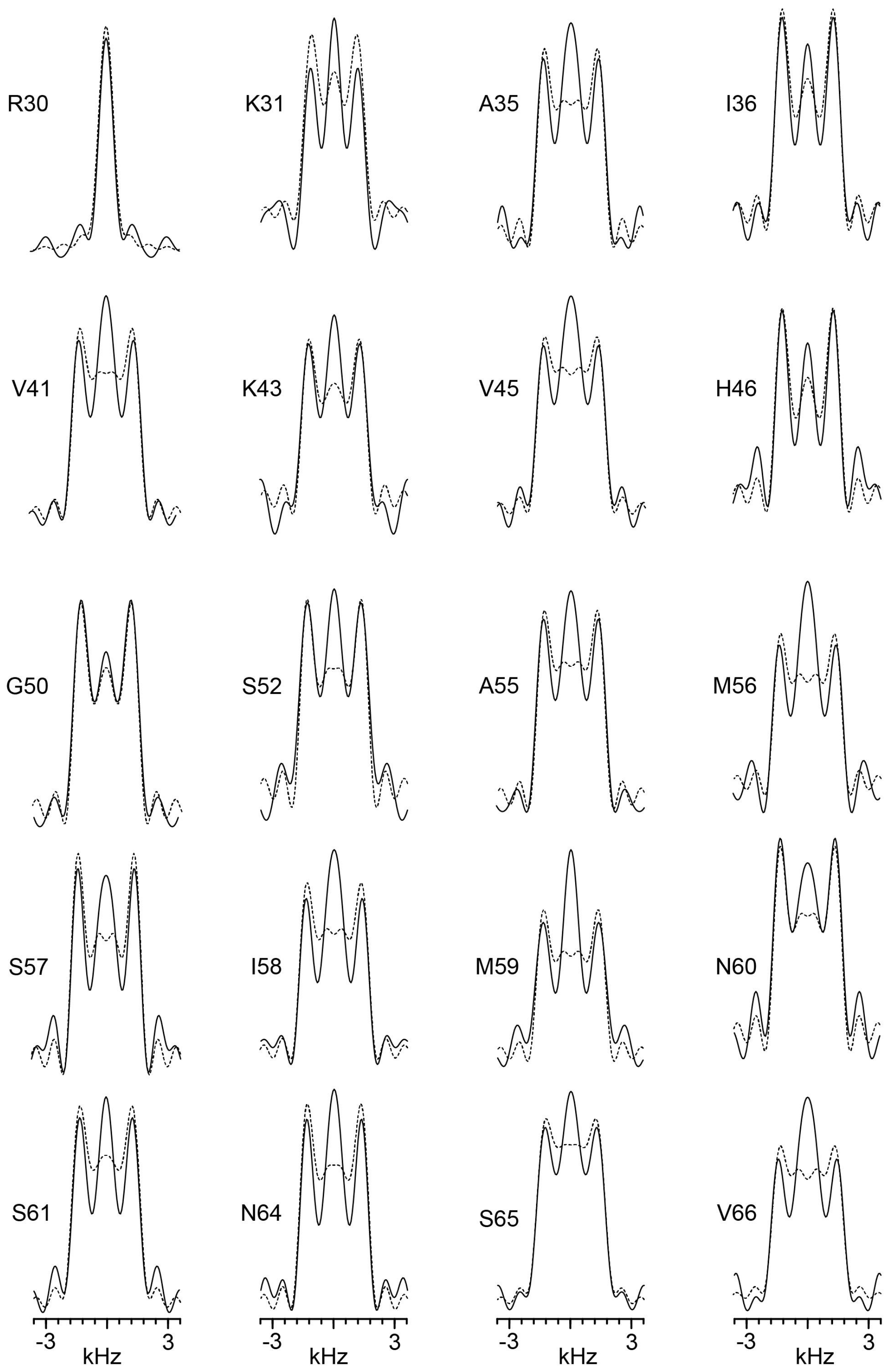

Fig. S5 Site-resolved experimental (solid) and simulated (dotted) ^{1}H-^{15}N dipolar line shapes for H2B in a Widom 601 NCP. Experimental line shapes are extracted from a 3D DIPSHIFT with $R12_1^4$ symmetry dipolar recoupling pulses. The simulated line shapes were obtained by using SIMPSON .

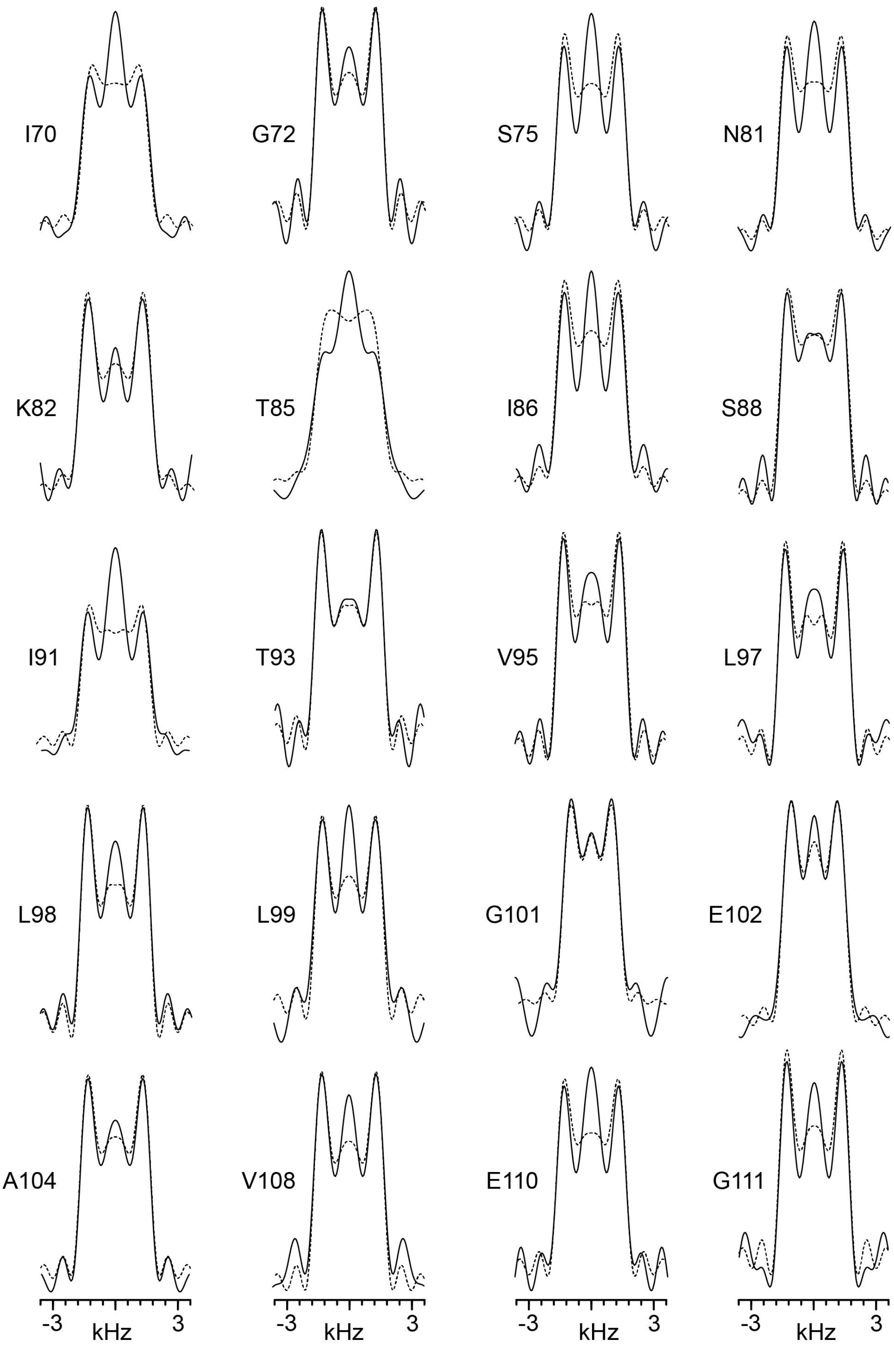

Fig. S5 (Continued)

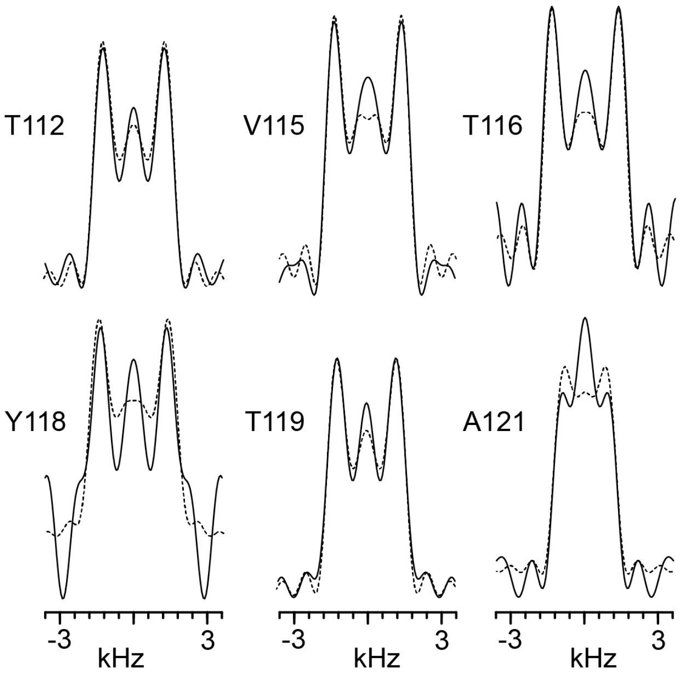

Fig. S5 (Continued)

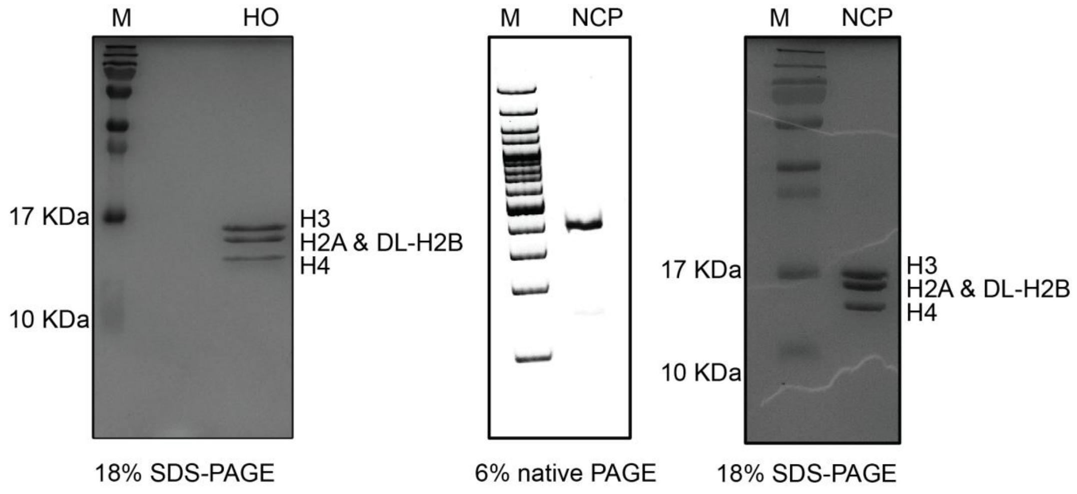

Fig. S6. Gel electrophoresis analysis of purified histone octamer (left panel) and reconstituted NCP (middle and right panels). Histones, H2A, H2B (^{13}C, ^{15}N double labeled), H3 and H4 in the histone octamer and the NCP are resolved on the 18% SDS-PAGE (M – Marker), and the quality of the NCP is further checked on the 6% native PAGE gel.
