# Supplementary material for: Structural and dynamical investigation of histone H2B in well-hydrated nucleosome core particles by solid-state NMR
Source: Commun Biol. 2023 Jun 24;6:672. doi: 10.1038/s42003-023-05050-3 (PMC10290710; doi:10.1038/s42003-023-05050-3)
Supplement: Supplementary file 2 — Description of Additional Supplementary Files [file 42003_2023_5050_MOESM2_ESM.pdf]

Description of Additional Supplementary Files

File name: Supplementary Data
Description: Data for making Figs. 2, 3 and 4.
